# Sleep Disturbances in Patients with Complex Regional Pain Syndrome: A Pilot Exploratory Study on Pain Characteristics, Functional Impairment, and Psychological Factors

**DOI:** 10.3390/brainsci16080874

**Published:** 2026-08-18

**Authors:** Iana Andreieva, Justyna Wiśniowska, Natalia Salata, Beata Tarnacka

**Affiliations:** 1 Department of Rehabilitation Medicine, Faculty of Medicine, Warsaw Medical University, Spartanska 1, 02-637 Warsaw, Poland; 2 Department of Rehabilitation, Eleonora Reicher National Institute of Geriatrics, Rheumatology and Rehabilitation, Spartanska 1, 02-637 Warsaw, Poland

**Keywords:** complex regional pain syndrome, sleep quality, daytime sleepiness, chronic pain, functional disability, central sensitization, pain catastrophizing

## Abstract

**Highlights:**

**What are the main findings?**

- Sleep disturbances affected over 80% of patients with complex regional pain syndrome and were strongly associated with affective and sensory pain severity.
- Reduced sleep duration and lower sleep efficiency were associated with higher pain intensity and neuropathic pain features.

**What are the implications of the main findings?**

- Routine assessment of sleep should be considered for incorporation into the multidisciplinary evaluation and management of patients with complex regional pain syndrome.
- Interventions targeting sleep quality may improve pain-related outcomes and functional recovery, warranting further investigation in larger prospective studies.

**Abstract:**

Background/Objectives: Our aim was to evaluate sleep quality and excessive daytime sleepiness in complex regional pain syndrome (CRPS) and explore their associations with pain characteristics, functional impairment, depressive symptoms, and pain coping strategies. Methods: This pilot exploratory, cross-sectional observational study was conducted at a single tertiary rehabilitation center using a convenience sample of 21 adults with CRPS Type I (n = 17) or Type II (n = 4). Sleep quality and daytime sleepiness were evaluated using the Pittsburgh Sleep Quality Index (PSQI) and Epworth Sleepiness Scale (ESS). Pain was assessed with the Numeric Rating Scale (NRS), Short-Form McGill Pain Questionnaire (SF-MPQ), and PainDETECT Questionnaire (PDQ). Limb-specific disability was measured using the Disability of the Arm, Shoulder and Hand (DASH) questionnaire for upper-extremity CRPS (n = 10) and the Lower Extremity Functional Scale (LEFS) for lower-extremity CRPS (n = 11). Psychological factors and central sensitization were assessed via BDI-II, CSQ, and CSI. Results: Poor sleep quality (PSQI > 5) occurred in 81.0% (17/21) of participants (mean PSQI: 10.1 ± 4.1). ESS-defined excessive daytime sleepiness was identified in 47.6% (10/21) of patients (mean ESS: 9.6 ± 5.8). In unadjusted bivariate correlation analyses of the full cohort (n = 21), global PSQI score significantly correlated with NRS pain intensity (ρ = 0.445, *p* = 0.0431), SF-MPQ sensory pain (ρ = 0.468, *p* = 0.0325), SF-MPQ affective pain (ρ = 0.461, *p* = 0.0354), and SF-MPQ total score (ρ = 0.504, *p* = 0.0199. Conclusions: Sleep disturbances and excessive daytime sleepiness are highly prevalent in CRPS patients in tertiary care. Exploratory unadjusted analyses link sleep quality with pain severity, particularly sensory and affective dimensions. Given the exploratory design and small subgroup sample sizes, larger prospective studies are needed to confirm these findings.

## 1. Introduction

Complex Regional Pain Syndrome (CRPS) is a chronic and debilitating pain condition characterized by severe, disproportionate pain typically arising after limb trauma, such as fractures or surgical procedures [1]. The disorder presents with a constellation of sensory, autonomic, vasomotor, and trophic disturbances, including altered skin temperature, edema, abnormal sweating, and changes in hair or nail growth. Symptoms usually develop within weeks of the inciting event and may persist long term, significantly impairing quality of life.

CRPS is more prevalent in women than in men and most commonly occurs in middle-aged and older adults. The upper extremities are affected more frequently than the lower extremities. Although the exact incidence varies across populations, CRPS remains a relatively rare but clinically significant condition [2].

The pathophysiology of CRPS is complex and multifactorial, involving interactions between peripheral inflammation, central sensitization, autonomic dysfunction, immune dysregulation, and maladaptive neuroplasticity [3]. These mechanisms create a self-reinforcing cycle of pain and functional impairment. In addition, psychosocial and environmental factors—such as exposure to stressful life events—may increase susceptibility to the condition. Established risk factors include female sex, specific types of fractures (e.g., distal radius), and a heightened pain response during the early post-injury phase [4].

CRPS is classified as a chronic primary pain condition, reflecting its systemic nature and its overlap with other pain syndromes such as fibromyalgia and chronic migraine. Importantly, chronic pain conditions, including CRPS, frequently coexist with sleep disturbances. A substantial proportion of individuals with chronic pain report impaired sleep, while sleep disorders themselves can predispose individuals to the development and persistence of pain [4].

The relationship between sleep and chronic pain is now understood to be complex and potentially bidirectional, although emerging evidence suggests that sleep disturbances may act as a stronger predictor of pain chronification than pain itself [5,6]. Disrupted sleep can influence pain perception through multiple biological pathways, including immune dysregulation, increased inflammatory mediators, and impaired endogenous pain inhibition. Neurobiological research further indicates that alterations in central neurotransmitter systems may simultaneously modulate both pain and wakefulness [7,8,9].

Although sleep disturbance is clinically recognized as a frequent and prominent symptom of CRPS, dedicated research on this issue remains remarkably scarce, with only a small number of studies available to date. This paucity of data is largely attributable to the low incidence and clinical rarity of CRPS, which inherently limits sample sizes in clinical research. Furthermore, in the majority of existing studies, sleep has been evaluated primarily in an indirect manner—often captured merely as a secondary sub-item within broad quality-of-life tools or reported simply as a basic prevalence rate [10,11,12].

While pre-existing sleep disturbances are increasingly recognized as important predictors of CRPS development and increased pain severity [13,14], comprehensive evaluations in CRPS are currently lacking. To date, there are no studies with assessment of distinct subjective sleep parameters—combining specific nocturnal sleep characteristics (such as latency and efficiency) and daytime sleepiness—alongside a detailed, multi-domain assessment of pain dimensions (sensory vs. affective), extremity-specific functional disability, central sensitization, and cognitive coping mechanisms. Moreover, the impact of sleep disruption on daytime functioning in CRPS has been addressed only indirectly, through general quality-of-life scores, rather than targeted measures of daytime sleepiness.

Therefore, this study aims to evaluate sleep quality and daytime sleepiness in patients with CRPS and to investigate their associations with pain characteristics, functional impairment, depressive symptoms, and pain coping strategies.

## 2. Materials and Methods

The pilot exploratory cross-sectional study was a one-group single-center study of 21 patients with CRPS Type I or Type II. This cross-sectional observational study was conducted in accordance with the STROBE reporting guidelines. The study was conducted from 10 October 2023 to 1 March 2025. The research protocol was approved by the Ethics Committee (Approval Number: KBT-4/2/2020). The research was completed in accordance with the Helsinki Declaration.

Patients were screened and diagnosed with CRPS according to the Budapest Criteria approved by the International Association for the Study of Pain (IASP) [15].

Inclusion criteria to study: subjects aged ≥ 18 years, diagnosed with CRPS, who were treated at the Department of Rehabilitation of National Geriatrics, Rheumatology and Rehabilitation Institute, named for prof. Eleonora Reicher, in Warsaw, Poland.

Subjects with pain conditions lacking the correct diagnosis code, symptoms not matching the Budapest criteria [15,16], or with insufficient information in the medical records were excluded. Others criteria of exclusion were: active drug or alcohol abuse within the last 12 months; active localized or systemic infections; major vascular diseases affecting the target limb, including acute deep vein thrombosis, severe peripheral vascular disease, or severe Raynaud’s phenomenon; systemic rheumatologic or inflammatory conditions (e.g., active rheumatoid arthritis, systemic lupus erythematosus) that could confound regional inflammatory or autonomic assessments; uncontrolled psychiatric comorbidities (e.g., major depressive disorder with suicidal ideation, schizophrenia, or bipolar disorder); current treatment with gabapentinoids, opiods, or antidepressants; and lack of informed consent.

Due to the inclusion criteria, all participants had to undergo a thorough medical examination before being admitted to the program. The physical medicine and rehabilitation doctor diagnosed the general condition of the CRPS limb.

In addition to the physical assessment, the patients were asked to complete a questionnaires. All returned questionnaires (n = 21) were analyzed.

A non-probability convenience sampling method was employed, enrolling all eligible consecutive patients meeting inclusion criteria during the recruitment period. No formal a priori sample size or statistical power calculation was conducted due to the exploratory nature of the study and the low epidemiological incidence of CRPS in clinical practice.

### 2.1. Functional Assessment

Validated functional assessment tools were incorporated to identify the life impact of CRPS on participants; these consisted of the Disabilities of the Arm, Shoulder and Hand outcome measurement tool (DASH) and Lower Extremity Functional Scale (LEFS) for the upper and lower limbs, respectively.

DASH Questionnaire: Evaluates upper-extremity disability, physical function, and symptom severity; score range is 0–100. Only core questionnaires were used; two optional modules (work and sports/performing arts) were not used to assess functional limitations in CRPS patients [17,18].

LEFS Questionnaire: Assesses functional impairment of the lower limbs during daily activities; score range is 0–80 [19].

### 2.2. Pain Assessment

Pain intensity and quality were assessed using the Numeric Rating Scale (NRS), the Short-Form McGill Pain Questionnaire (SF-MPQ), and PainDETECT questionnaire (PD-Q).

SF-MPQ is a brief self-report instrument designed to assess the sensory and affective dimensions of pain. SF-MPQ A (Sensory Subscale) evaluates the sensory–discriminative dimension of pain across 11 descriptors (items 1–11: throbbing, shooting, stabbing, sharp, cramping, gnawing, hot–burning, aching, heavy, tender, splitting). Each item is scored on a 4-point intensity scale, yielding a sum score with a theoretically possible range of 0 to 33. SF-MPQ B (Affective Subscale) evaluates the affective–motivational dimension of pain across 4 descriptors (items 12–15: tiring–exhausting, sickening, fearful, punishing–cruel), scored on the same 0–3 intensity scale, yielding a sum score with a theoretically possible range of 0 to 12 [20].

Neuropathic pain components were screened using the PainDETECT questionnaire (PD-Q). It generates a score from 0 to 38. Responses are scored to classify pain as likely neuropathic (total score ≥ 19), possibly mixed, or likely non-neuropathic (score ≤ 12) [21].

Central sensitization was assessed using the Central Sensitization Inventory (CSI), a 25-item self-report questionnaire that evaluates the presence and severity of symptoms associated with central sensitivity syndromes. It assesses somatic, emotional, and sleep-related symptoms (part A). Responses were scored from 0 (never) to 4 (always), with a total score of 40 or higher indicating the presence of central sensitization. Only Part A was used for scoring [22].

### 2.3. Sleep Assessment

Two different sleep assessment tools were used. The Pittsburgh Sleep Quality Index (PSQI) is a 19-item self-report questionnaire that was used for the assessment of sleep quality over the last month, with an overall global score above five indicating poor quality of sleep; sleep duration was also captured [23]. A global score > 5 indicates clinically significant sleep disturbance. Individual component scores were also evaluated, including sleep onset latency (where subscores of 2–3 indicate moderate-to-severe latency prolongation) and sleep efficiency (calculated as total sleep time divided by time spent in bed, expressed as a percentage).

Daytime sleepiness was assessed using the Epworth Sleepiness Scale (ESS), an 8-item self-report questionnaire. Total scores range from 0 to 24, with a score of 10 or higher interpreted as excessive daytime sleepiness [24].

### 2.4. Psychological Assessment

Depression assessment was measured with the Beck Depression Inventory-II (BDI-II) score [25]. The BDI-II is a self-reported questionnaire assessing depression severity, with possible scores ranging from 0 (no depression) to 66 (severe depression).

Pain-coping strategies were evaluated using the Polish adaptation of the Coping Strategies Questionnaire (CSQ; Juczyński, 2001) [26]. Following standard psychometric validation of the Polish CSQ adaptation, we analyzed three composite higher-order domain factors: Cognitive Coping, Diverting Attention and Resalting, and Catastrophizing. Subscale scores range from 0 (strategy not engaged) to 36 (strategy significantly engaged), with higher scores indicating greater deployment of the respective coping mechanism [26].

### 2.5. Statistical Analysis

Descriptive statistics were calculated for all study variables and are presented as means and standard deviations (SDs) for continuous variables, together with medians, interquartile ranges (IQRs), and minimum and maximum values where appropriate. Categorical variables were summarized using frequencies and percentages. The distribution of continuous variables was assessed visually using histograms and Q–Q plots and evaluated with the Shapiro–Wilk test.

Differences in continuous demographic, pain (NRS, SF-MPQ, PDQ), functional (DASH, LEFS), and psychological (BDI-II, CSI, CSQ) outcomes between subgroups were analyzed using the Mann–Whitney U test. Categorical variables were compared using Fisher’s exact test.

Associations were examined using Spearman rank correlations. Statistical significance was set at *p* = 0.05. Due to the low incidence of CRPS and the resulting small cohort (n = 21), all statistical analyses were conducted as exploratory bivariate correlations to assess pattern trends and effect sizes, rather than confirmatory inferential modeling.

All statistical analyses were conducted using SPSS 27.0 (IBM Corp., Armonk, NY, USA).

Missing data were handled using a complete-case analysis approach without imputation. It was restricted to limb-specific functional questionnaires, which were administered exclusively based on the anatomical site of CRPS involvement: the DASH questionnaire was completed by the 10 patients with upper-extremity CRPS, while the LEFS questionnaire was completed by the 11 patients with lower-extremity CRPS.

## 3. Results

**Figure 1:**
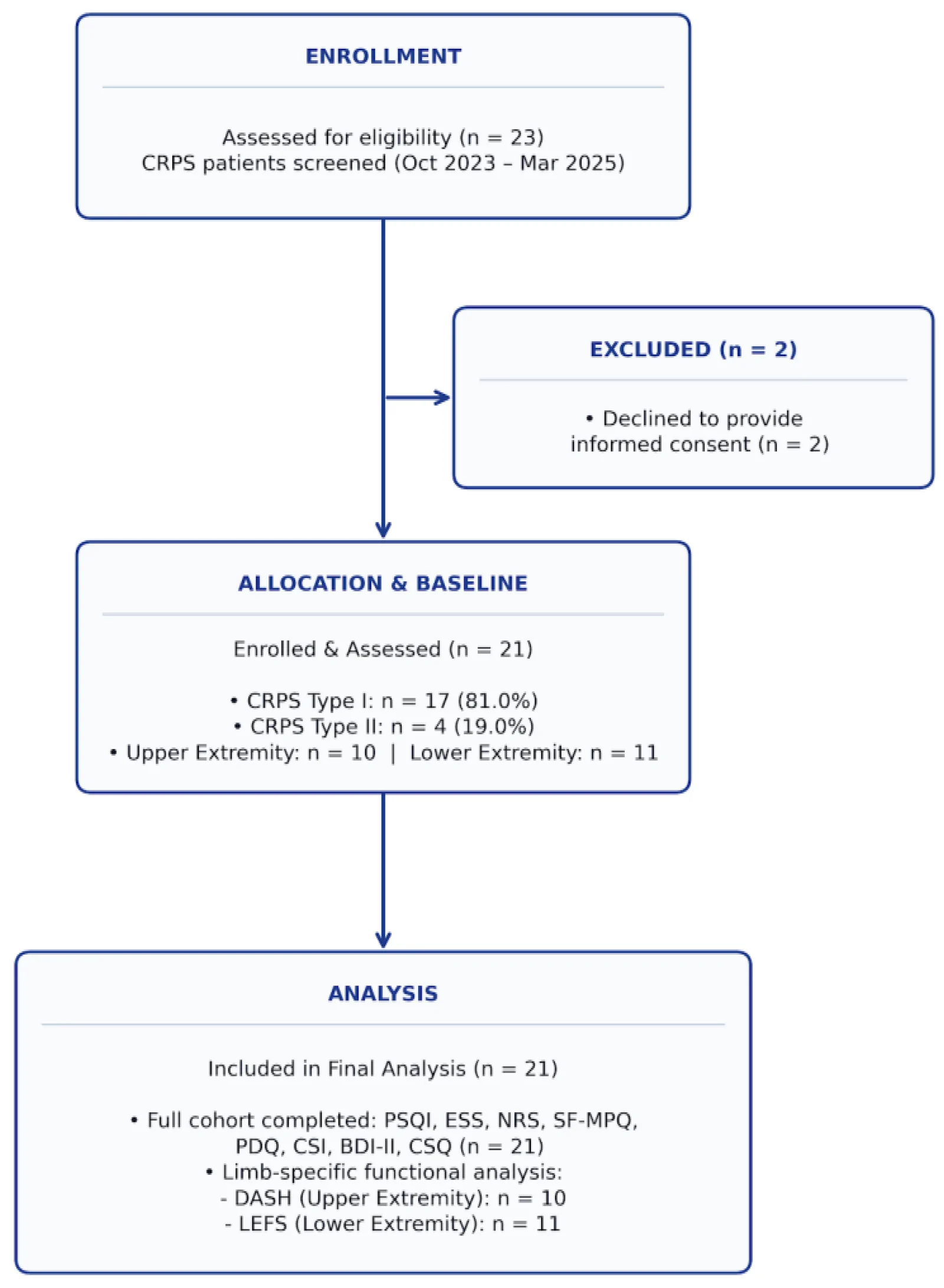
Participant flow diagram.

A total of 23 patients with CRPS were screened for eligibility between October 2023 and March 2025. Two patients were excluded prior to enrollment because they did not provide written informed consent. Consequently, 21 patients met all inclusion criteria, provided informed consent, and completed the evaluation. All 21 enrolled participants were included in the final data analysis. The complete flow of participants through the study is illustrated in Figure 1.

Seventeen of the participants met criteria for CRPS-I, four had a diagnosis of CRPS-II. The general demographic and clinical variables are presented in Table 1.

Among participants who developed CRPS following an injury, 76.2% (16/21) had sustained fractures, including one open fracture (4.7%, 1/21). When surgical intervention was required, fracture stabilization was performed using either permanent fixation devices (14.3%, 3/21) or temporary metalwork fixation (19.1%, 6/21). The wrist and forearm were the most frequently reported fracture sites (47.6%, 10/21), followed by the foot (28.6%, 6/21).

The median time to symptom development was 19 days (7–42 days). Patients received formal diagnoses via Budapest criteria in 2.6 months (1.1–4.8 months) after the inciting events. Patients were included in the study at a median of 5.2 months (2.1–8.5 months) after CRPS symptom onset.

Before CRPS development, only three patients reported temporary sleep disorders; one patient had insomnia, and one patient had treatment with Zolpidone before development of CRPS.

A total of 21 patients provided complete data on sleep quality and pain measures (Table 2).

The mean global PSQI score was 10.1 ± 4.1, exceeding commonly used clinical cut-offs for poor sleep quality. A total of 17/21 patients (81.0%) had PSQI scores above the clinical cut-off. The average sleep duration was 6.33 ± 1.37 h, while the average time spent in bed was 8.02 ± 1.33 h. Sleep efficiency reached an average of 79.39 ± 12.24%, indicating reduced sleep quality. The mean sleep latency was 38.00 ± 22.68 min, suggesting prolonged sleep onset latency in the studied group.

More than half of the patients (52%) reported no use of sleep medications, indicating that hypnotic use is not common in this group. However, a notable proportion of patients (48%) reported some level of use, with four patients using sleep medication at least once per week (scores 2–3). A smaller subgroup (three patients) reported frequent use (≥3 times per week), which may suggest a higher dependence on pharmacological sleep support.

On the PSQI categorical latency component, 71.4% (n = 15/21) presented with moderate-to-severe sleep onset disturbance (scores 2 or 3).

The mean Epworth Sleepiness Scale (ESS) score for the cohort was 9.6 ± 5.8. However, 10 out of 21 participants (47.6%) demonstrated an ESS score higher than 10.

To evaluate differences related to daytime sleepiness, participants were dichotomized into normal daytime sleepiness (ESS < 10, n = 11) and excessive daytime sleepiness (ESS > 10, n = 10) subgroups. Patients with excessive daytime sleepiness demonstrated significantly higher levels of CSI compared to those with normal sleepiness (median CSI: 35.0 [IQR: 33.2–48.5] vs. 27.0 [IQR: 18.5–33.0], Mann–Whitney (U = 24.0, *p* = 0.031). Additionally, a trend toward higher depressive symptoms was observed in the ESS > 10 group (median BDI-II: 12.0 [IQR: 9.0–18.8] vs. 8.0 [IQR: 4.5–11.5], U = 28.5, *p* = 0.066). No statistically significant differences were observed between the two groups regarding pain intensity (NRS), neuropathic symptoms (PDQ), global PSQI score, or pain coping strategies (*p* > 0.20 for all) in our study.

The mean DASH score (n = 10) was 57.7 ± 19.2 points, indicating a moderate to severe level of upper limb disability in the CRPS group. Most patients reported noticeable functional limitations. Only a small subgroup presented with relatively mild disability.

The mean LEFS (n = 11) score was 50.6 ± 16.0, reflecting a moderate level of lower limb functional capacity. While some patients achieved higher scores (above 70), others scored below 40.

Pain intensity was elevated across all applied measures. In the analyzed group, the highest mean score was observed for the SFMPQ-B scale (58.7 ± 27.7), accompanied by a notably wide range of values (10–100).

The CSI-A suggested a moderate level of variability across participants (33.1 ± 12.9). Among the assessed measures, SF-MPQ-B exhibited the greatest dispersion.

The average PDQ score (17.7 ± 5.3) was consistent with a moderate degree of neuropathic pain-related symptoms. Based on PDQ scores, 11 of 21 participants (52.4%) had likely neuropathic pain (PDQ ≥ 19), five participants (23.8%) had a possible mixed pain mechanism (PDQ 13–18), and five participants (23.8%) were classified as having likely non-neuropathic pain (PDQ ≤ 12). Thus, over half of the sample demonstrated a neuropathic pain profile according to PDQ classification criteria. The distribution was relatively homogeneous compared with CSI-A, as reflected by the smaller standard deviation.

The average BDI score (11.48 ± 6.79) suggests that, on average, patients presented with mild depressive symptoms, although substantial variability was observed across individuals.

Results of the psychological assessment are shown in Table 3.

According to the results of analysis, several associations were identified between sleep quality and pain-related measures. In non-parametric bivariate correlation analyses, the primary endpoint of pain intensity (NRS) demonstrated a moderate, statistically significant positive association with poor sleep quality as measured by the PSQI Total Score (ρ = 0.445, 95% CI: 0.046 to 0.751, *p* = 0.0431). Functional disability metrics showed moderate point correlations with PSQI (ρ = 0.506 for DASH and ρ = −0.208 for LEFS); however, these associations did not reach statistical significance (*p* = 0.1355 and *p* = 0.5397, respectively).

Among the exploratory endpoints, multidimensional pain characteristics evaluated by SF-MPQ were significantly correlated with higher PSQI scores. PSQI exhibited positive correlations with the SF-MPQ Sensory subscale (ρ = 0.468, 95% CI: 0.027 to 0.790, *p* = 0.0325), SF-MPQ Affective subscale (ρ = 0.461, 95% CI: 0.056 to 0.727, *p* = 0.0354), and the SF-MPQ Total Score (ρ = 0.504, 95% CI: 0.111 to 0.768, *p* = 0.0199).

Psychological metrics (BDI depression: ρ = 0.284; CSQ Catastrophizing/Hope: ρ = 0.351) and central sensitization (CSI: ρ = 0.286) showed trend-level positive associations with sleep disturbance (PSQI). Detailed correlation metrics are presented in Table 4.

No statistically significant associations were found between CSI scores and global sleep quality measures at the r = 0.05 significance level.

For central sensitization and psychological metrics, correlations with global sleep quality (PSQI) were non-significant and remain inconclusive (Table 4).

A positive correlation was observed between BDI scores and CSQ_KATA (ρ = 0.832, 95% CI: 0.062 to 0.930, *p* < 0.001). Cognitive coping strategies tended to be associated with lower depression. The factor of cognitive coping score showed a moderate negative correlation (ρ = −0.352, 95% CI: −0.686 to, 0.100, *p* = 0.118). No significant associations were identified between depressive symptoms and the remaining CSQ coping dimensions (all *p* > 0.05). Pain control showed a trend toward a negative association with depression. The moderate trend association was observed between BDI and CSI (ρ = 0.386, 95% CI: −0.059 to 0.705, *p* = 0.084). Only weak-to-moderate correlations were found between depression and pain measures: NRS (ρ = 0.180), PainDetect (ρ = 0.222), SF-MPQ Sensory (ρ = 0.248), SF-MPQ Total (ρ = 0.320). No meaningful association was observed between BDI and daytime sleepiness measured by ESS. Additional analyses demonstrated that catastrophizing was positively associated with central sensitization (ρ = 0.493, 95% CI: 0.076 to 0.765, *p* = 0.023). The coping strategies evaluated by the CSQ were not associated with daytime sleepiness or pain characteristics in the studied cohort.

## 4. Discussion

Patients with CRPS very frequently have sleep disorders, and research suggests a strong bidirectional relationship between CRPS-related pain and sleep.

Our findings reveal a high prevalence of sleep disturbance within the CRPS patient population according to results of PSQI. Our findings are consistent with those reported in similar studies. Galer et al. (2000) [10] reported that the majority of CRPS patients suffered from sleep disturbance. In a large-scale, multicenter study by Lee et al. (2021) [12], showing an exceptionally high prevalence of sleep disturbances (92.1% of patients) and extremely short sleep duration (4.92 h), there was a high proportion of patients with long-standing disease in their sample. Specifically, more than half of the participants had been undergoing treatment for over 3 years, and 11.7% for more than 10 years.

In another study by Lee et al. (2014) [13], where the mean disease duration was 2.84 years, poor sleep quality was reported in 97% of patients. Furthermore, when analyzing risk factors for suicidal ideation, no significant difference in disease duration was found between the high-risk and low-risk groups, although insomnia itself emerged as a significant correlate. Patients from our study group with higher depression scores tended to report poorer sleep quality, although not statistically significant. The direction of the association is consistent with the extensive literature demonstrating reciprocal interactions between depression and sleep impairment in chronic pain populations. Hill et al. (2012) [27] argued that CRPS is associated with depression, anxiety, and insomnia; however, the relationship is directional, rather than psychopathological. The authors further stated that there is no evidence that psychological factors cause the onset of pain in CRPS and noted that pain and related discomfort can interfere with sleep onset and sleep maintenance.

No statistically significant correlation was observed between depressive symptoms (BDI) and sleep quality disturbances (PSQI). Although previous studies have frequently reported significant associations between depression and sleep disturbances, the current findings do not support this relationship.

Similarly, no meaningful association was found between depressive symptoms and daytime sleepiness as assessed by the ESS. This finding suggests that, within the present sample, the severity of depressive symptoms was not related to subjective daytime sleepiness. A finding of our study is that patients with excessive daytime sleepiness demonstrated significantly elevated levels of CSI compared to those with normal daytime sleepiness (39.8 ± 13.6 vs. 27.1 ± 9.0, *p* = 0.031). Subjective daytime sleepiness often indicates altered sleep architecture, reduced delta-wave sleep, or persistent nocturnal awakening.

To the best of our knowledge, no studies have investigated daytime sleepiness in patients with CRPS.

Poor sleep quality and daytime dysfunction correlated most strongly with affective and sensory pain dimensions, rather than pain intensity alone. In CRPS, altered central pain processing, autonomic dysfunction, and stress-related neurochemical changes likely contribute to both heightened pain and disrupted sleep, forming a “pain–sleep cycle” where each exacerbates the other [9].

Contrary to expectations, central sensitization was not significantly associated with sleep quality. Given the known role of central sensitization in pain amplification, further investigation is warranted. A randomized, sham-controlled trial of J.Vanhanen (2023) [28] found moderate correlations between pain interference and insomnia severity. We did not identify any additional studies that similarly evaluated pain and sleep disturbances.

Moderate correlation was observed between BDI and CSI; although not statistically significant, the effect size is moderate and suggests that patients with more severe depressive symptoms tended to report greater manifestations of central sensitization. Depression and central sensitization are believed to share common neurobiological mechanisms involving altered pain modulation, enhanced nociceptive processing, and emotional distress.

The results of correlation analysis between depressive symptoms and catastrophizing suggest that depressive symptoms were more strongly related to how patients cognitively processed pain (catastrophizing) than to pain intensity itself. Patients who catastrophized pain more frequently also exhibited significantly higher levels of central sensitization symptoms.

The absence of significant correlations between pain coping strategies assessed by the CSQ and measures of sleep quality (PSQI, ESS), pain severity (SF-MPQ), and neuropathic pain symptoms (PDQ) may have several explanations. The coping strategies represent relatively stable cognitive and behavioral responses to pain, rather than direct indicators of pain intensity or sleep disturbances. Variables such as disease duration, medication use, pain chronicity, anxiety, and emotional distress may exert stronger influences on sleep quality and pain perception than coping strategies alone. Interestingly, despite the lack of associations with sleep and pain measures, catastrophizing demonstrated a strong positive correlation with depressive symptoms. This finding is consistent with previous research indicating that catastrophizing primarily reflects emotional distress and maladaptive cognitive appraisal of pain, rather than pain intensity itself. A comprehensive cohort study following patients living with CRPS reveals that long-term CRPS patients exhibit high rates of pain catastrophizing, which correlates directly with an increased severity of physical symptoms, worse overall upper-limb disability, and diminished life satisfaction [4].

Sleep disturbances were also significantly associated with upper extremity dysfunction, but not lower extremity function. This discrepancy may reflect differences in functional demands, but there was no comparison analysis of functional subgroups in our study. In a study by Gudrun-Karin Kindl et al. (2024) [29], CRPS patients had a similar mean DASH (M = 54.7, S.D. ± 21) as brachial plexus lesion patients (M = 51.4, S.D. ± 16.1), but differed significantly from fracture controls (M = 21.2, S.D. ± 21.1). Pain and older age were predictors of the DASH in this study [29].

Overall, the results emphasize the clinical importance of addressing sleep disturbances in patients with CRPS. Interventions targeting sleep quality—particularly those aimed at improving sleep efficiency and reducing sleep latency—may have beneficial effects on pain perception and functional outcomes. Future studies with larger samples are needed to clarify the role of central sensitization and to identify independent predictors of sleep impairment in this population.

### Limitation

The relatively small sample size (n = 21) limited statistical power and increased the risk of Type II error, potentially explaining why several clinically meaningful associations did not reach statistical significance. Sleep quality was evaluated exclusively using self-reported questionnaires (PSQI and ESS), without objective measurements such as polysomnography or actigraphy. The study was conducted at a single center, which may limit the generalizability of the findings to broader CRPS populations. Participants recruited from a specialized pain clinic may represent individuals with greater symptom severity or longer disease duration compared to community-based CRPS populations. Functional assessment required different instruments depending on the affected limb, reducing the possibility of direct comparison between upper- and lower-extremity CRPS. Medication use, including analgesics, antidepressants, anticonvulsants, and hypnotics, was not controlled for in the statistical analyses and may have influenced both sleep and pain outcomes. Future longitudinal studies with larger samples and objective sleep assessments are required to confirm these findings and better characterize the mechanisms linking sleep disturbances with pain, psychological factors, and functional disability in CRPS.

## 5. Conclusions

Sleep disturbances were highly prevalent in this cohort of adults with CRPS. Subjective sleep impairment and excessive daytime sleepiness are highly prevalent in patients with CRPS. In our cohort (n = 21), poor overall sleep quality (PSQI > 5) occurred in 81.0% (17/21) of patients, while excessive daytime sleepiness (ESS > 10) was identified in 47.6% (10/21) of participants. Unadjusted bivariate analyses indicated that reduced sleep duration and lower sleep efficiency were associated with greater pain intensity and neuropathic symptoms.

These findings highlight the importance of assessing sleep in clinical evaluations, though larger prospective studies are required to establish whether independent relationships exist between psychological factors, central sensitization, and sleep impairment in CRPS.

## Figures and Tables

**Table 1:** The general demographic and clinical characteristics of the patients.

Characteristic	CRPS Cohort (n = 21)
Age, (years)	52.7 ± 7.9
Sex, n (%):	
Female, n (%)	11 (52.4%)
Male, n (%)	10 (47.6%)
CRPS Classification, n (%):	
Type I (RSD), n (%)	17 (80.9%)
Type II (Causalgia), n (%)	4 (19.1%)
Affected Site, n (%):	
Upper Extremity	10 (47.6%)
Lower Extremity	11 (52.4%)
Involved Side, n (%):	
Dominant Limb	12 (57.1%)
Non-Dominant Limb	9 (42.9%)
Primary Inciting Event, n (%):	
Fracture	16 (76.2%)
Sprain/Contusion	4 (19.0%)
Spontaneous	1 (4.8%)
Treatment, n (%):	
Surgical treatment	9 (42.9%)
Non-surgical treatment	12 (57.1%)
Symptoms onset, (days)	19 days (7–42 days)
Time to Diagnosis, (months)	2.6 months (1.1–4.8 months)
Disease Duration at Assessment, (months)	5.2 months (2.1–8.5 months)
Medications, n (%):	
Non-Steroidal Anti-Inflammatory Drugs (monotherapy)	9 (42.9%)
Active Hypnotic/Sedative Use,	1 (4.8%)
Current/Recent Corticosteroid Therapy (Systemic)	5 (23.8%)
Prior Temporary Sleep Disturbances, n (%)	3 (14.3%)
Pre-existing Diagnosed Insomnia, n(%)	1 (4.8%)

**Table 2:** Results of patients’ assessments.

Measure	Mean ± SD/ Median [Q1–Q3]	Range [Min–Max]	n
PSQI global	10.1 ± 4.1	1–17	21
Epworth (ESS)	9.6 ± 5.8	1–21	21
NRS pain	5.00 [4.00–8.00]	3–8	21
PDQ	17.7 ± 5.3	10–29	21
SF-MPQ A	18.0 ± 9.0	4–36	21
SF-MPQ B	58.7 ± 27.7	10–100	21
CSI	33.1 ± 12.9	17–65	21
DASH	57.7 ± 19.2	5–86.7	10
LEFS	50.6 ± 16.0	21–74	11

**Table 3:** Results of the psychological assessment.

Variable	Mean ± SD	Range [Min–Max]	n
Catastrophizing (CSQ_KATA)	11.57 ± 7.53	0–29	21
Attention Diversion/Replacement Activities Factor	31.90 ± 15.54	0–51	21
Cognitive Coping Strategies Factor	45.67 ± 18.60	14–83	21
Beck Depression Inventory (BDI)	11.48 ± 6.79	1–24	21

**Table 4:** Summary of correlation analysis with PSQI.

Clinical/Psychological Parameter	Spearman ρ	95% Bootstrap CI	Raw *p*-Value	n
Pain Intensity (NRS)	0.445	[0.046, 0.751]	0.0431	21
DASH	0.506	[−0.196, 0.861]	0.1355	10
LEFS	−0.208	[−0.700, 0.423]	0.5397	11
SF-MPQ Sensory Subscale	0.468	[0.027, 0.790]	0.0325	21
SF-MPQ Affective Subscale	0.461	[0.056, 0.727]	0.0354	21
SF-MPQ Total Score	0.504	[0.111, 0.768]	0.0199	21
Central Sensitization (CSI Baseline)	0.286	[−0.132, 0.638]	0.2090	21
Depression (BDI Baseline)	0.284	[−0.190, 0.690]	0.2128	21
CSQ Catastrophizing/Hope	0.351	[−0.117, 0.740]	0.1188	21
CSQ Distraction/Action	−0.068	[−0.520, 0.421]	0.7689	21
CSQ Cognitive Coping	−0.026	[−0.400, 0.341]	0.9124	21
Daytime Sleepiness (ESS Baseline)	0.105	[−0.316, 0.544]	0.6510	21

## Data Availability

Due to privacy and ethical restrictions regarding participant confidentiality, the raw datasets cannot be made publicly available. However, deidentified individual participant data, the data dictionary, and the analysis syntax will be shared upon reasonable request to the corresponding author. Access to deidentified data may require a formal data-sharing agreement and approval from the local Ethics Committee. Aggregate data are fully reported within the manuscript.

## Abbreviations

The following abbreviations are used in this manuscript:

CRPS	Complex regional pain syndrome
PSQI	Pittsburgh Sleep Quality Index
ESS	Epworth Sleepiness Scale
NRS	Numeric Rating Scale
SF-MPQ	Short-Form McGill Pain Questionnaire
PDQ	PainDETECT Questionnaire
DASH	Disabilities of the Arm, Shoulder and Hand
LEFS	Lower Extremity Functional Scale
CSI	Central Sensitization Inventory
BDI-II	Beck Depression Inventory-II
CSQ	Coping Strategies Questionnaire
CSQ_KATA	CSQ-Catastrophizing
